# Intimate partner violence among pregnant women attending antenatal care services in the rural Gambia

**DOI:** 10.1371/journal.pone.0255723

**Published:** 2021-08-05

**Authors:** Joseph W. Jatta, Ararso Baru, Olufunmilayo I. Fawole, Oladosu A. Ojengbede

**Affiliations:** 1 Institute of Life and Earth Science (Including Health and Agriculture), Pan African University, Ibadan, Nigeria; 2 Slum and Rural Health Initiative Network, Research and Collaboration Department, SRHIN/Africa, Ibadan, Nigeria; 3 College of Medicine and Health Sciences, Arbaminch University, Arbaminch, Ethiopia; 4 Department of Epidemiology and Medical Statistics, Faculty of Public Health, College of Medicine, University of Ibadan, Ibadan, Nigeria; 5 Centre for Population and Reproductive Health, College of Medicine, University of Ibadan, Ibadan, Nigeria; Xi’an Jiaotong University School of Medicine, CHINA

## Abstract

**Background:**

Intimate partner violence (IPV) refers to any behavior by either a current or ex-intimate partner or would-be rejected lover that causes physical, sexual, or psychological harm. It is the most common form of violence in women’s lives. According to a World Health Organization report, about 1 in 3 women worldwide experience at least one form of IPV from an intimate partner at some point in her life. In the Gambia, about 62% of pregnant women experience at least one form of violence from an intimate partner. IPV has severe physical and mental health consequences on a woman ranging from minor bodily injury to death. It also increases the risk of low birth weight, premature delivery, and neonatal death.

**Methods:**

A health facility-based cross-sectional study design was carried out to assess the magnitude and factors associated with intimate partner violence among pregnant women seeking antenatal care in the rural Gambia. The study enrolled 373 pregnant women, and a multi-stage sampling technique was used to select the respondents. An interviewer-administered structured questionnaire was used to obtain information from the study participants. The collected data were analyzed using SPSS Ver.22. Bivariate and multivariate logistic regression were used to determine the association between dependent and independent variables. Odds ratio with 95% confidence interval (CI) was computed to determine the presence and strength of associated factors with IPV.

**Result:**

The study reveals that the prevalence of IPV in The Gambia is 67%, with psychological violence (43%) being the most common form of IPV reported by the respondents. The multivariate logistic regression result reveals that being aged 35 years or older [AOR 5.1(95% CI 1.5–17.8)], the experience of parents quarreling during childhood [AOR 1.7(95% CI 1.0–2.75)], and having cigarette smoking partners [AOR 2.3 (95% CI 1.10–4.6)] were significantly associated with IPV during pregnancy.

**Conclusion:**

This study has demonstrated that all forms of IPV in rural Gambia are frequent. Women older than 35 years, had experienced parents quarreling, had a partner who smoked, and a partner who fight with others were more likely report IPV compared to other pregnant women in the study. We recommend that IPV screening should be included as an integral part of routine antenatal care services in The Gambia. Community-based interventions that include indigenous leaders, religious leaders, and other key stakeholders are crucial to create awareness on all forms of IPV and address the risk factors found to influence the occurrence of IPV in rural Gambia.

## Background

Intimate partner violence (IPV) is a severe social, medical, and public health issue in all societies [1]. IPV refers to any behavior by either a current or ex-intimate partner or would-be rejected lover that causes physical, sexual, or psychological harm. It includes, but is not limited to, physical aggression, sexual coercion, controlling behavior, psychological, cultural, spiritual, and financial abuse [2].

Evidence suggests that IPV is the most common form of violence in women’s lives [3]. According to a World Health Organization’s (WHO) report, about 1 in 3 women worldwide experience at least one form of IPV from an intimate partner at some point in her life [4]. A review of literature on sub-Saharan Africa (SSA) reveals that the reported IPV among pregnant women by their intimate partners varies from as low as 2% to as high as 66.9% [5–8].

Similarly, IPV is a serious social and health problem in the Gambia [9, 10]. According to a population based study conducted in The Gambia, over 40% of women reported at least one form of IPV [11]. The burden of IPV is even much higher among pregnant women; a previous study conducted in The Gambia indicates that 61.8% of pregnant women experienced at least one form of violence from an intimate partner [9].

IPV has dramatic physical and mental health consequences on the woman, ranging from minor physical injury to death. Stab, gunshot, and head injuries are among the most common physical consequences experienced by pregnant women worldwide [12]. The mental health consequences include depression, suicide ideations, insomnia and low self-esteem [1, 13]. Moreover, IPV has also an adverse effect on fetal health. It increases the risk of low birth weight, premature delivery, and neonatal death [14, 15].

The high prevalence of IPV among pregnant women is worrisome, and some of the factors associated with IPV include, place of residence, educational status, partner’s age, age at sexual debut, dowry payment, substance abuse by husband, and history of violence in the family [16–18].

Addressing the factors associated with IPV among pregnant women in a given society is a crucial step toward preventing IPV and enhancing maternal and child health. As a global partner in gender-based violence (GBV) control, the main goal of The Gambia’s National GBV control program has been the reduction of the burden of GBV by 2020 in line with the Millennium development goals and recently Sustainable Development Goals (SDGs) [19, 20].

Although The Gambia has a high burden of IPV [9, 10], there is a dearth of literature on the factors associated with IPV among pregnant women in the rural Gambia to the best of our knowledge. The available literature on domestic violence in The Gambia focuses mainly on sexual violence among commercial sex workers [10] and physical violence against women [21]. A study focused on intimate partner violence against pregnant women in The Gambia; however, the study was conducted in a single center and particularly restricted to IPV among pregnant women in the urban setting [9]. As a result, available information in the Gambia in general and the rural Gambia, in particular, remained limited. Thus, identifying prevalence and factors associated with intimate partner violence in rural Gambia could be one of the critical steps to minimize the prevalence of IPV and associated adverse maternal and child health outcomes. So, this current study investigates the prevalence and factors associated with IPV among pregnant women in the rural Gambia.

## Methods

### Study setting and design

This study was conducted at the Upper River Region (URR) of The Gambia. URR is one of the five administrative regions in the country. People in this region, along with the Central River Region (CRR), have the lowest ratings on social indicators than other areas [22]. Each region in the country has a health team that a regional director heads. Under each region, there are hospitals and primary health care facilities, which include major and minor health centers, public and private clinics. The primary health care facilities provide health services to people of the communities in its catchment areas. URR has 12 health centers, and all women seeking antenatal care services at six selected health centers were included in the study. The selected health centres are as follows: Basse Health Centre, Garawol Health Centre, Baja Kunda Health Centre, Demba Kunda Health Centre, Koina Health Centre, and Yorobawol Health Centre.

A health facility-based cross-sectional study design was employed to assess the magnitude and the factors associated with intimate partner violence among pregnant women seeking antenatal care at selected public health facilities in the rural Gambia from February 1 to May 15, 2019.

### Eligibility criteria

All pregnant women live within the rural setting for the past six months, and volunteers to participate in the study were included in the study. The respondents who were critically ill or who were in labor were excluded from the study as there were ethical and moral challenges in recruiting such respondents in the study.

### Sample size determination

Single population proportion formula was used to estimate sample size:

$$n=\frac{{\left[Z(\alpha )\right]}^{2}XP(q)}{d^{2}}$$

Where:

n = Minimum sample size for a statistically significant survey

Z = Standard normal deviate at 95% confidence interval two-tailed test is; = 1.96

P = Indicates prevalence of IPV among pregnant women which was 61.8% and taken from the previous study [9].

q = 1-p, d = margin of error taken as 5%= 0.05

After substituting into the formula, the computed sample size was 363. The final sample size with a 10% assumption for non-response rate was 400.

### Sampling procedure

A multi-stage sampling technique was used to select respondents.

Stage 1: The health facilities in URR were stratified into two strata based on their functions, namely: major and minor health centers. For the major health centers, they were four, and by simple balloting, 3 out of the four were selected, with each health center attending to an average of 60 antenatal women per antenatal clinic day. The same procedure was followed for the eight remaining minor health centers, and three were eventually selected. The specific sample size was allocated to each stratum using proportion-to-size allocation.

Stage 2: A sampling frame was created from the registry at the antenatal care entry point. The systematic random sampling technique was used to select respondents, which was determined by dividing the estimated average number of women who attended ANC of each facility in a previous similar period for the allocated sample size for each health facility. Lastly, a respondent was selected by every K^th^ number of pregnant women coming to each health facility for ANC service.

### Data collection tool and techniques

An interviewer-administered structured questionnaire was used to collect information from each participant. We adapted a questionnaire that WHO initially developed for a multi-country study on women’s health, which we modified to fit both the objectives of our research and the culture of the respondents [23]. Self-reported experience of IPV during the current pregnancy was the main outcome of this study. The IPV assessed in this study included all physical, sexual, psychological, and economic forms of violence. To identify sexual violence, each woman was asked a series of four questions. To confirm physical violence, six questions were asked; to assess psychological violence, eleven questions were asked; two questions were asked to identify economic violence. The details on these questions are given in Table 1.

**Table 1:** A series of questions asked to confirm various forms of IPV among pregnant women in the rural Gambia.

**Psychological violence or Controlling behavior**	**Does your partner try to keep you from seeing your friends?**
	Does your partner try to keep you from seeing your family of birth?
	Does your partner insist on knowing where you are all the time?
	Does your partner ignored you and treated you indifferently?
	Does your partner get angry if you speak with another man?
	Is your partner often suspicious that you might be unfaithful?
	Does he expect you to ask for his permission before you seek healthcare for yourself?
	Does he insult you or make you feel bad about yourself?
	Does he belittle or humiliate you in front of other people?
	Has he ever done things to scare or intimidate you on purpose during the current pregnancy (e.g. the way he looks at you, by yelling or smashing things)?
	Does he threaten to hurt you or people you care about?
**Physical violence**	Does he slap you or threw something at you that could hurt you?
	Does he push or shove or pull your hair?
	Does he hit you with his fist or with an object that could hurt you?
	Does he kick or drag or beat you up?
	Does he threaten to use a gun or knife or some other weapons against you?
	Does he choke or burn you on purpose?
**Sexual violence**	Does your partner physically force you to have sexual intercourse against your interest/will?
	Have you ever had sexual intercourse with him because you were afraid of what he might do to you?
	Has he ever forced you to do something sexually that you found degrading or humiliating?
	Does he refuse to have sex to hurt you?
**Economic violence**	Does your partner deny you money or other material things to hurt you?
	Does he refuse to let you work or do any form of business?

### Operational definitions

#### Intimate partner

A husband, cohabiting partner, boyfriend, or lover, or ex-husband, ex-partner, ex-boyfriend, or ex-lover [24].

#### Intimate partner violence

Refers to behavior by an intimate partner that causes physical, sexual or psychological devastation, including actions of physical violence, sexual intimidation, emotional abuse, and controlling behaviors [24].

### Data quality assurance

The data quality was assured through careful design, translation, and retranslation of study tool language from the English version to the national languages (Mandinka, Serahuli, and Wolof) and vice versa. The questionnaire was pretested before data collection, and possible corrections were made based on the feedback from the pretest. Participants were approached and interviewed during ANC visits by the language of their preference. In addition, two days of training were given to the data collectors and supervisors. Furthermore, continuous and close supervision of the data collecting procedures, proper categorization, and coding of the data was done. JWJ and AB checked the completeness and consistency of the data daily.

### Data entry, processing, and analysis

Data were checked for completeness and inconsistencies, and were cleaned, coded, and entered into the EPI data. Statistical Package for Social Science (SPSS) version 22 was used for the analysis.

Frequency tables, diagrams, and proportions were used to present and summarize the variables of interest. Logistic regression was used to determine the degree of relationship between dependent variables and their associated factors. Evidence has shown that selecting a variable with a p-value cut off 0.05 as a candidate for multivariate analysis can fail in identifying variables known to be important [25]. On the contrary, a purposive selection of a cutoff value of 0.25 has the capability of retaining both significant covariates and important confounding variables in logistic regression [26]. Similarly, a cut-off value of 0.25 has been supported by different studies [25, 27]. Therefore, this study considered all variables with a p-value of < 0.25 on bivariate analysis for multivariate logistic regression analysis, and statistical significance was set at a 5% level.

### Ethical approval and consent to participate

Ethical approval was obtained from the Ethical Review Board of the University College of Medicine, the University of Ibadan in Nigeria. Furthermore, ethical clearance was obtained from the Research and Publication Committee of the University of the Gambia (RePUBLIC) and then The Gambia Government/Medical Research Council Joint Ethics Committee (Reference number: R018031V1.1) for permission to conduct this study. The study followed the National Institute of Health guidelines on research involving the use of human subjects.

Permission to conduct the study in the URR Health Region was sought and obtained from the Regional Health Team. Furthermore, the research’s aim, purpose, and importance were clearly and in unambiguous terms explained to each respondent to obtain informed verbal consent. Therefore, each respondent signed a pre-designed consent form in the interviewer’s presence to endorse their participation.

Confidentiality of the information obtained from each participant was maintained. All the data and information collected were coded using numbers, and not at any one point in time was the name of a single participant known. The information on subjects was accessible to only the principal investigator and entered into a password-protected computer.

## Results

This study included a total of 373 women in the analysis, making a response rate of 93.3% as twenty-seven women were not included in the analysis because of incomplete data.

### Sociodemographic characteristics of respondents

Table 2 shows that most of the respondents are between the age group of 20–24 years, followed by those in the age group of 25–29 years, which accounted for 27% and 26% of the study participants, respectively. About two-thirds of respondents are non-formally educated (66%), and only 2% have attained tertiary education. The vast majority are married (97%), and Islam is practiced by 98% of them. Eighty-seven percent were housewives, of whom about 10% are self-employed, and 3% reported having paid employment. The distribution by tribe shows that 39% (the majority) of the respondents are Serahuli, followed by Mandinka (29%).

**Table 2:** Sociodemographic characteristics of the pregnant women seeking antenatal service at public health care facilities in the rural Gambia, 2019.

Variables	Frequency (n = 373)	Percentage (%)
**Age Group (years)**		
18–19	59	15.8
20–24	100	26.8
25–29	98	26.3
30–34	66	17.7
35–39	24	6.4
40+	7	1.9
Undisclosed	19	5.1
**Level of Education**		
No formal	245	65.7
Arabic	14	3.8
Primary education	72	19.3
Secondary education	33	8.8
Tertiary education	9	2.4
**Marital Status**		
Single	3	0.8
Married	363	97.3
Divorced	7	1.9
**Religion**		
Christianity	2	0.5
Islam	365	97.9
Traditionalist	6	1.6
**Occupation**		
Housewife (unemployed)	324	86.9
Paid employment	13	3.5
Self-employed	36	9.7
**Tribe**		
Serahuli	147	39.4
Mandinka	110	29.5
Fula	97	26.0
Wollof	10	2.7
Others were	9	2.4

### Partners sociodemographic status and lifestyle

Table 3 shows that the majority (29%) of the respondents have partners in the age range of 30–39 years. Half of the women’s partners have no formal education (50%), while only 10% reportedly attained tertiary education. Almost all the women (99%) have partners who practice Islam. The majority (81%) of the women’s partners are self-employed, and 16% have paid employment.

**Table 3:** Sociodemographic and lifestyle characteristics of partners of pregnant women seeking antenatal service at public health care facilities in the rural Gambia, 2019.

Variables	Frequency (n = 373)	Percentage (%)
**Age Group (years)**		
20–29	105	28.2
30–39	108	29.0
40–49	61	16.4
50+ years	31	8.3
Do not know	68	18.2
**Level of Education**		
No formal	188	50.4
Arabic	38	10.2
Primary education	40	10.7
Secondary education	68	18.2
Tertiary education	39	10.5
**Religion**		
Christianity	1	0.3
Islam	371	99.4
Traditionalist	1	0.3
**Occupation**		
Unemployed	11	2.9
Paid employment	60	16.1
Self-employed	302	81.0
**Partner’s Tribe**		
Serahuli	131	35.1
Mandinka	114	30.6
Fula	108	29.0
Wollof	11	2.9
Others	9	2.4
**Partner’s Current Cigarette Smoking Status**		
Never	305	81.8
Rarely	28	7.5
Moderately	17	4.6
Heavily	23	6.2
**Partner’s Current Alcohol Drinking Status**		
Never	367	98.4
Yes	2	1.6
**History of Previous Engagement in Fights**		
Yes	87	23.3
No	205	55.0
Don’t know	81	21.7

About 22% of the women’s partners are habitual cigarette smokers, while 6% are reported to be heavy smokers. Only 2% of the partners are reported to engage in drinking alcohol. Twenty-three percent of the women said their partners had been previously involved in fights with other persons, while 55% claimed their partners have never engaged in fights with others.

### Experience of intimate partner violence

The prevalence of intimate partner violence is 67%, as revealed in Table 4; thus, indicating that 67% of the women have experienced at least one form of IPV.

**Table 4:** Prevalence of intimate partner violence among pregnant women seeking antenatal services at public health care facilities in the rural Gambia, 2019.

Type of violence	Frequency (n = 373)	Percentage (%)
**Psychological violence**	239	64.1
Expects you to ask for his permission before you seek healthcare for yourself	114	30.6
Insists on always knowing where you are	111	29.8
Gets angry when you speak with another man	103	27.6
He tries to keep you from seeing your friends	67	18.0
Insults you or makes you feel bad about yourself	52	13.9
He is often suspicious that you are unfaithful	51	13.7
Ignores you and treats you indifferently	36	9.7
Belittles or humiliates you in front of other people	35	9.4
Do things on purpose scare or intimidate you (e.g. the way he looks at you, by yelling or smashing things)	23	6.2
Tries to restrict contact with your family of birth	22	5.9
Threatens to hurt you or someone you care about	15	4.0
**Physical violence**	59	15.8
Slaps you or throws thing at you that could hurt you	45	12.1
Pushes you or shoves you or pulls your hair	15	4.0
Hits you with his fist or with objects that could hurt you	15	4.0
Kicks you, drags you or beats you up	14	3.8
Threatens to use a gun or used a gun, knife or other weapons against you	5	1.3
Chokes and burns you on purpose	4	1.1
**Sexual violence**	27	7.2
Have sexual intercourse with him because you are afraid of what he might do to you	17	4.6
Physically forces you to have sexual intercourse when you do not want to	14	3.8
Refuses to have sex with you in other to hurt you	11	2.9
Forces you to do something sexually that you find degrading or humiliating	4	1.1
**Economic violence**	40	10.7
Denies you money or other material things to hurt you	26	7.0
Refuses to let you work or do any form of business	20	5.4
**The overall experience of IPV**	250	67.0

### Disaggregation of the prevalence of intimate partner violence

**Fig 1:**
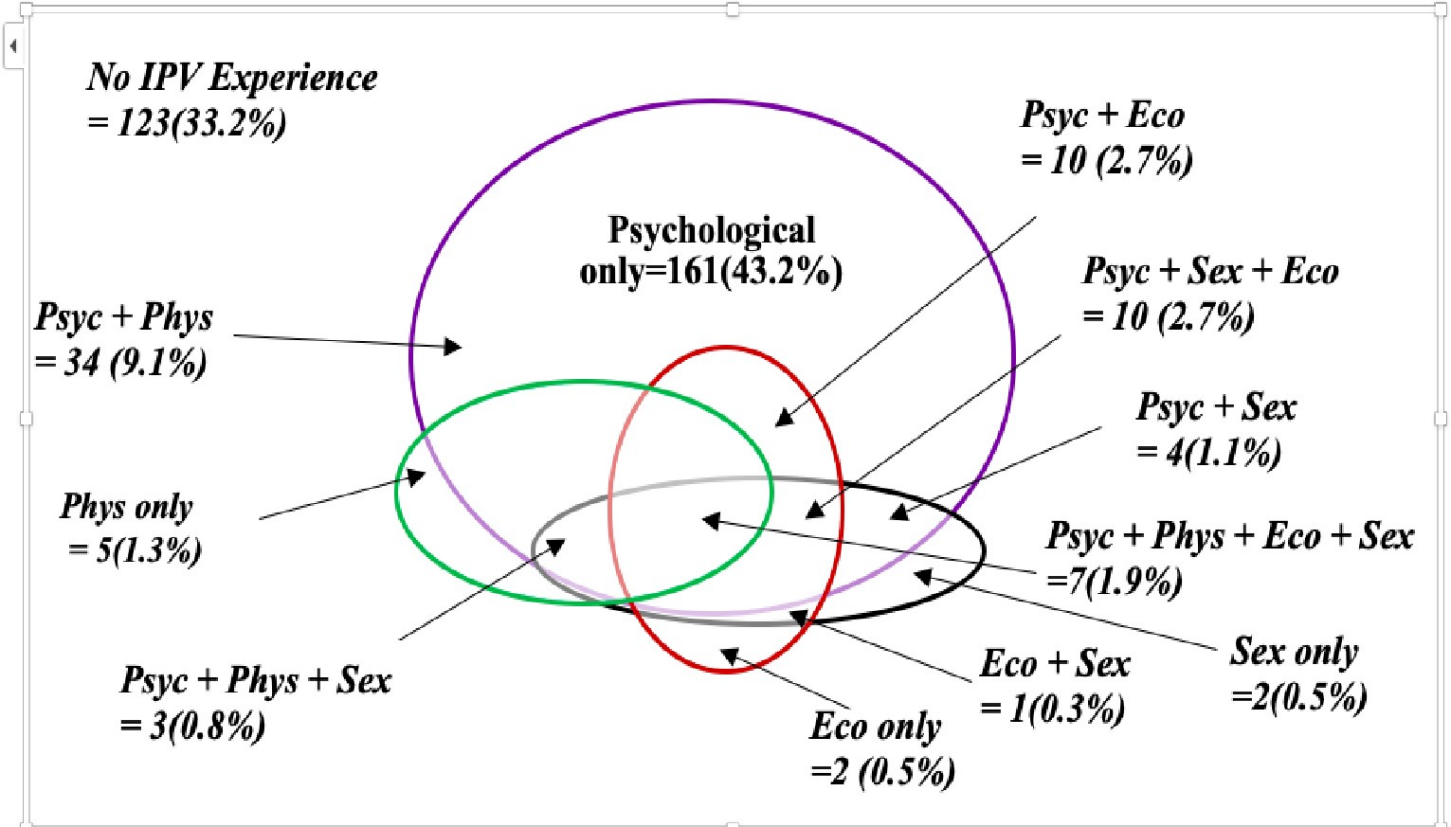
The overlapping forms of IPV perpetrated against pregnant women in the rural Gambia. Note: N = 373; Psyc = psychological; Phys = physical; Eco = economic; sex = sexual.

Further disaggregation of the prevalence of intimate partner violence as revealed in Fig 1 exposes the following: while 33% had never experienced any form of violence from their partners, 43% had experienced only the psychological violence, about 9% reported having experienced the combination of psychological and physical violence, nearly 3% reported experiencing a combination of psychological, sexual and economic violence, while almost another 3% had experienced a combination of psychological, physical & economic violence, and about 2% reported experiencing all forms of violence.

### Factors associated with intimate partner violence

Factors found to be associated with intimate partner violence are presented in Table 5. It is found that women of ages 35 years and above are about five times more likely to have had an IPV experience than those in the age group 18–24 years. The women’s experience of witnessing a quarrel between their parents is associated with their IPV experience. The odds ratio estimated from the logistic regression shows that women who had seen their parents quarrel are almost twice likely to have experienced IPV. Women with partners who smoke have two and a half more chances of experiencing IPV. Women who reported having witnessed their partners engage in a fight are found to have twice more chances of experiencing any form of IPV than those who have never seen their partners involved in a fight (OR = 2.05, p = 0.010).

**Table 5:** Factors associated with IPV among pregnant women in rural Gambia, 2019.

Variables	Experienced IPV		COR 95% CI	AOR 95% CI
	Yes	No		
**Age Group (in years)**				
18–24	64	95	Ref.	Ref.
25–34	61	103	1.14(0.80–1.90)	1.2(0.73–1.9)
≥35	4	27	4.45(1.70–19.93)	5.1(1.5–17.8)
**Witnessed Parents Quarrelling**				
No	96	137	Ref.	Ref.
Yes	38	102	1.96(1.23–3.13)	1.7(1.0–2.75)
**Partner Smoking Status**				
No	120	85	Ref.	Ref.
Yes	14	54	3.05(1.54–6.056)	2.3(1.10–4.6)
**Witnessed Partner Fight**				
No/Don’t know	21	66	Ref.	Ref.
Yes	113	173	2.10(1.183–3.644)	1.7(1.89–3.10)

## Discussion

This study seeks to determine the prevalence and factors associated with IPV among a sample of pregnant women in the rural Gambia. The study findings show that various forms of violence, namely psychological, sexual, physical, and economic violence, were experienced by pregnant women attending ANC services in the rural Gambia.

Our findings reveal that 67% of the pregnant women in this study experienced at least one form of violence from their intimate partner, comparable with similar studies from Ethiopia and Kenya, which reported 68.6% and 66.9% prevalence, respectively [18, 28]. On the other hand, our finding is slightly higher than the report of a similar study in The Gambia, which reports a 61.8% prevalence of IPV [9]. The disparity could be because the previous research from The Gambia only assessed three forms of IPV. In comparison, the present study assessed more than three forms of IPV, including economic violence. In addition, the previous study was conducted in the urban Gambia compared to the present study, which was conducted in the rural Gambia [9]. It has been reported that rural women experience higher IPV rates than urban women [29].

This study also assesses various forms of IPV and their overlapping experienced by pregnant women. The study shows that the most common form of violence experienced by pregnant women was psychological violence alone (43.2%). Even though the reported prevalence from Rwanda was relatively lower than that of the present study (31.7%), psychological violence alone was the most common form of violence in the study [30]. The difference could be attributable to cultural differences between the two countries, wherein in the Gambia, men are considered to be in charge of women; for example, permission must be sought and granted before the woman can go out, or a good woman obeys her husband even if she disagrees with him. These are societal norms in rural Gambia. We have also observed that the most common overlapping IPV among pregnant women is psychological and physical violence. Similar findings were presented from Tanzania and Rwanda [30, 31].

The second most experienced IPV among the respondents is physical violence (15.8%), which is much lower than that of the previous study from The Gambia, which reported a 55% prevalence of physical IPV [9]. A possibility of underreporting among the women in the rural Gambia cannot be ruled out, which may be due to denial, shame, or stigma associated with violence in a marital relationship. Therefore, further studies are needed to justify the reasons for the observed differences. On the other hand, a study from Nigeria reports a 17.3% prevalence of IPV among pregnant women, a figure close to the findings in this study [32]. This could be attributed to similarities in religious and traditional beliefs between the two countries.

The findings of this study indicate that sexual violence is reported by 7.2% of the respondents, which is comparable to the study conducted in Bolivia, which reports 6.9% [33]. However, a cross-sectional study in Ethiopia reports a 36% prevalence of sexual violence among female respondents, which is much higher than the finding of this study [34]. The extreme disparity could be attributed to the fact that talking about the subject matter (sexual experience) is highly sensitive in the Gambia [9]. Therefore, the chances of disclosing if they are experiencing some form of sexual violence from their intimate partners are very slim.

On the other hand, more than 10% of the respondents reported having experienced economic violence from their intimate partners during the current pregnancy. Although the study from Ghana assesses the lifetime prevalence of economic violence, their finding is similar to that of the present study [35]. This similarity can be attributed to the fact that most women in this part of the world depend entirely on their husbands for financial support [36, 37]. This can be seen in the demographic data, which indicates that over 85% of the respondents in this present study are housewives. Moreover, the controlling behavior tendencies by men could ensure that some prevent their wives from engaging in any money-making venture [36].

In this study, a woman’s age is significantly associated with IPV as all the women aged 35 or older are five times likelier to experience IPV from their partners. This can be compared to a study from Sweden, which also finds age as a significantly associated factor to experiencing intimate partner violence [13]. This study also demonstrates an association between a partner who smokes and intimate partner violence among pregnant women in the rural Gambia. A previous study from The Gambia also finds a similar association between having a partner who smokes and IPV experience [9]. The explanation could be that, pregnancy influence the association between IPV and cigarette smoking as there are higher baseline levels of physical and emotional distress that place pregnant women at greater risk of victimization compared to non-pregnant women [38].

The present study finds out that those women who witnessed a quarrel between their parents during childhood were twice likelier to suffer IPV than other women who did not. These findings are consistent with a comparable study from Brazil, which reports that the women who witnessed their mothers quarrelling suffer from IPV than their counterparts who did not witness such [39]. The social learning theory postulates that children learn to exhibit aggressive behaviors because they observe others acting aggressively and can see how these behaviors are reinforced over time [40]. Violence, therefore, tends to have an impact on children who then, in turn, grow to be either perpetrators or victims of IPV. Moreover, the present study finds out that women who witnessed their husbands engage in a fight with other people were twice likelier to experience IPV. This could be explained as the partners being generally aggressive and quarrelsome, they do not spare anyone of their quarrels, including their wives.

## Limitations of the study

This study has some limitations. First of all, the data used in this study were collected using self-reported experiences. As a result, due to the sensitive nature of the subject matter in the rural Gambia, social desirability bias may have led to underreporting of IPV in general and sexual IPV. Secondly, the study’s findings could be affected by a recall bias as respondents were required to remember and recount past events. When respondents remember past events, they don’t usually have a complete or accurate picture of what happened. Moreover, a causal relationship would have been challenging to establish due to the study design implemented in the study, which was a cross-sectional study. Therefore, the findings of this study should be considered in light of the limitations above.

## Conclusion

This study has demonstrated that all forms of IPV in the rural Gambia are frequent, with psychological violence found to be the most highly prevalent. The study finding illustrates that being older than 35 years, having a cigarette smoking partner, experienced parent quarreling during childhood, and witnessing partners fight with others were the factors that significantly associated with IPV experience during the current pregnancy.

We recommend that IPV screening should be included as an integral part of routine antenatal care services in The Gambia. Community-based interventions that include indigenous leaders, religious leaders, and other key stakeholders are crucial to create awareness on all forms of IPV and address the risk factors found to influence the occurrence of IPV in the rural Gambia. More research is required to provide information from male partners’ side, associated factors of IPV, and effects of IPV on the outcome of pregnancy in The Gambia.

## Supporting information

S1 Appendix (DOCX)

## Abbreviation

AB: Ararso Baru
AOR: Adjusted odds ratio
ANC: Antenatal care
COR: Crude odds ratio
CRR: Central River Region
GBV: Gender-based Violence
IPV: Intimate partner violence
JWJ: Joseph W. Jatta
OAO: Oladosu A. Ojengbede
OIF: Olufunmilayo I. Fawole
PAULESI: Pan African University Institute of Life and Earth Science Including Health and agriculture
RePUBLIC: Research and Publication Committee of the University of the Gambia
SSA: Sub-Saharan Africa
SPSS: Statistical Package for Social Sciences
URR: Upper River Region
WHO: World Health Organisation
