# Supplementary material for: Intimate partner violence among pregnant women attending antenatal care services in the rural Gambia
Source: PLoS One. 2021 Aug 5;16(8):e0255723. doi: 10.1371/journal.pone.0255723 (PMC8341542; doi:10.1371/journal.pone.0255723)
Supplement: S1 Appendix — (DOCX) [file pone.0255723.s001.docx]

**S1 APPENDIX**

**Prevalence and factors associated with intimate partner violence among pregnant women and their partners in rural Gambia.**

My name is_____________. I am conducting a survey on naval personnel partner’s health and life experiences. Please answer these questions honestly and sincerely as your responses will guide decisions to be made. I assure you that all your answers will be kept strictly secret and confidential. No record of your name address is required; neither will any information on your partner’s name and contact be required. Also no information given will be used against your partner. You have the right to discontinue from participating in the any point in time and can skip any question you don’t want to answer. However, your honest respond will be appreciated as your experience could be helpful to other women. Thank you in anticipation of your cooperation in answering the questions.

SERIAL NO ………………..

**SECTION 1: SOCIO-DEMOGRAPHIC DATA**

1. Sex [ ] 1 Male [ ] 2 Female
2. Age in years (age as at last birthday) [ ]
3. Highest level of education [ ] 1 None [ ] 2 Primary [ ] 3 Secondary [ ] 4 Tertiary
4. Tribe [ ________________________________]
5. Religion [ ] 1 Christianity [ ] 2 Islam [ ] 3 Traditionalist [ ] 4 Others
6. Occupation [ ] 1 House wife (None) [ ] 2 Paid Employment [ ] 3 Self Employed
7. Do you currently smoke cigarette? [ ] 1 Yes [ ] 2 No
8. If yes to question no. 6, on average how many stick per day [ ] 1 Less than 5 [ ] 2 5 – 10 [ ] 3 above 10
9. Do you take alcoholic drinks? [ ] 1 Yes [ ] 2 No
10. If yes in question no.9, on the average what is your intake? [ ] 1 Everyday/Nearly every day [ ] 2 Once or a Twice a week [ ] 3 1 – 3 times in a month [ ] 4 Occasionally to less than once a month.
11. Do you occasionally take drugs (e.g marijuana, cocaine, heroin etc) [ ] 1 Yes [ ] 2 No
12. Did you as a child sometimes see your parents quarrelling or fighting? [ ] 1 Yes [ ] 2 No
13. What is your partner’s age in years? (as at last birthday) [ ]
14. Your partners rank. [ ] 1 Rating [ ] 2 Officer
15. What is your partner’s highest level of education? [ ] 1 School Certificate [ ] 2 Graduate [ ] 3 Postgraduate
16. What is your partner Tribe? [ ] 1 Fula 2 [ ] Mandinka 3 [ ] Wollof [ ] 4 Others
17. What is your partner Religion? [ ] 1 Christianity [ ] 2 Islam [ ] Traditionalist [ ] 4 Others
18. Does your partner currently smoke cigarette? [ ] 1 Never [ ] 2 Rarely [ ] 3 Moderately [ ] 4 Heavily
19. Does your husband take drinks containing alcohol? [ ] 1 Never [ ] 2 Rarely [ ] 3 Moderately [ ] 4 Heavily
20. Since you met your partner, has he been involved in a physical fight with another man/woman? [ ] 1 Yes [ ] 2 No [ ] 3 Don’t know/ Don’t remember

**SECTION 2: EXPERIENCE OF INTIMATE PARTNER VIOLENCE**

Please you are required to tick Yes or No to que3stion no. 21 and if you tick Yes, indicate the number of times the action occurred during the current pregnancy

1. Has your husband/partner done any of the following to you?

|  |  | YES | NO | If yes, how many times in the last 12months? | | |
| --- | --- | --- | --- | --- | --- | --- |
|  |  |  |  | Once or Twice | A few (3-5) times | Many (more than 5)times |
| a | Tried to keep you from seeing your friends |  |  |  |  |  |
| b | Tried to restrict contact with your family of birth |  |  |  |  |  |
| c | Insisted on knowing where you are at all times |  |  |  |  |  |
| d | Ignored you and treated you indifferently |  |  |  |  |  |
| e | Gets angry if you speak with another man |  |  |  |  |  |
| f | Is often suspicious that you are unfaithful |  |  |  |  |  |
| g | Expected you to ask for his permission before you seek healthcare for yourself |  |  |  |  |  |
| h | Insulted you or made you feel bad about yourself |  |  |  |  |  |
| I | Belittled or humiliated you in front of other people |  |  |  |  |  |
| J | Did things to scare or intimidate you on purpose (e.g. the way he looks at you, by yelling or smashing things) |  |  |  |  |  |
| k | Threatened to hurt you or someone at you care about |  |  |  |  |  |
| L | Slapped you or threw something at you that could hurt you |  |  |  |  |  |
| m | Pushed you or shoved or pulled your hair |  |  |  |  |  |
| n | Hit you with his fist or with object that could hurt you |  |  |  |  |  |
| o | Kicked you, dragged you or beat you up |  |  |  |  |  |
| p | Threatened to use a gun or actually used a gun, knife or other weapon against you |  |  |  |  |  |
| q | Physically forced you to have sexual intercourse when you didn’t want to |  |  |  |  |  |
| R | Choked and burned you on purpose |  |  |  |  |  |
| S | Denied you money or other material things in order to hurt you |  |  |  |  |  |
| T | Had sexual intercourse with him because you were afraid of what he might do to you |  |  |  |  |  |
| u | Forced you to do something sexually that you found degrading or humiliating |  |  |  |  |  |
| v | Refused to have sex with you in other to hurt you |  |  |  |  |  |
| w | Refused to let you work or do any form of business |  |  |  |  |  |

**SECTION 3: HISTORY OF CHILDHOOD ABUSE**

Please you are required to answer YES or NO to question no. 22, and if yes indicate the number of items it occurred.

1. Before you were 15years, did you experience any of the following? \

|  |  | Yes | No | If yes how many times did it occur before your 15^th^ birthday | | |
| --- | --- | --- | --- | --- | --- | --- |
|  |  |  |  | Once or twice | A few (3-5) times | Many (more than 5) times |
| a | Were you severely beaten by your parents or guardian when you are was a child? (< 15yrs) |  |  |  |  |  |
| b | Did any man naked himself or masturbated before you when you were a child? (< 15 yrs) |  |  |  |  |  |
| c | Did any man fondle you or have sexual penetration with you? (< 15yrs) |  |  |  |  |  |

THANK YOU FOR YOUR TIME
